# Factors related to the use of opioids as early treatment in patients with knee osteoarthritis

**DOI:** 10.1186/s13075-019-2004-x

**Published:** 2019-11-04

**Authors:** Soo-Kyung Cho, Sun-Young Jung, Seongmi Choi, Seul Gi Im, Hyoungyoung Kim, Woo Seok Choi, Eun Jin Jang, Yoon-Kyoung Sung

**Affiliations:** 1 Department of Rheumatology, Hanyang University Hospital for Rheumatic Diseases, 222-1 wangsimni-ro, Seongdong-gu, Seoul, 04763 Republic of Korea; 2 College of Pharmacy, Chung-Ang University, 84, Heukseok-ro, Dongjak-gu, Seoul, 06974 Republic of Korea; 3 Department of Statistics, Kyungpook National University, 80, Daehak-ro, Buk-gu, Daegu, 41566 Republic of Korea; 4 Department of Information Statistics, Andong National University, 1375, Gyeongdong-ro, Andong-si, Gyeongsangbuk-do 36729 Republic of Korea

**Keywords:** Osteoarthritis, Knee, Opioids, Treatment

## Abstract

**Objective:**

To examine factors related to the use of opioids as an early treatment option for knee OA patients

**Methods:**

Using the Korean nationwide claim database, we selected knee OA patients between 2013 and 2015. Among them, patients without any claim of knee OA for 2 years before the index date were included as our study population. We analyzed their first claim for prescriptions, including tramadol and stronger opioids, at the index date of each patient. Using a multinomial model, we identified factors associated with the early use of tramadol and stronger opioids in knee OA patients.

**Results:**

Among a total of 2,857,999 knee OA patients, 12.2% (*n* = 348,516) were treated with opioids as their first treatment. However, the prevalence of stronger opioid use was only 0.07% (*n* = 1972). Male sex (OR 1.28 in tramadol, OR 1.13 in stronger opioids) and comorbidities with depression (OR 1.05, 1.46), low back pain (OR 1.13, 1.30), intervertebral disc disorder (OR 1.11, 1.40), and spinal stenosis (OR 1.27, 1.55) were the factors for the early use of tramadol or stronger opioids in knee OA patients. Patients in a tertiary referral hospital tended to use tramadol or stronger opioids than those in clinics (OR 1.04, 56.63, respectively).

**Conclusion:**

In Korea, 12.2% of knee OA patients were treated with opioids as an early treatment, and tramadol was used more commonly than stronger opioids. Male sex and having comorbidities such as depression or musculoskeletal disease are patient factors associated with the early use of opioids in knee OA patients.

## Introduction

Osteoarthritis (OA) is a highly prevalent, chronic condition affecting nearly 25% of adults aged 55 years and older in the United States (US) [1], and its prevalence continues to rise [2]. Knee OA is one of the most frequent diseases around the world, with more than 250 million sufferers [3]. In Korea, a recent report described the number of knee OA patients aged more than 50 years in males and females as 21.1% and 43.8%, respectively [4].

Current guidelines for knee OA treatment recommend a range of non-pharmacological and pharmacological interventions to alleviate pain and improve function and quality of life. Most guidelines do not recommend opioids for knee OA treatment as an early treatment option [5, 6], but some guidelines suggest using opioids on a restricted basis for short-term use in patients with refractory symptoms [7]. However, a recent Cochrane systematic review of 22 randomized controlled trials showed that opioids yielded a small clinical benefit, but with a high risk of side effects, including opioid dependence in patients with hip and knee OA [8]. Furthermore, a recent study suggested that preoperative opioid use in knee OA patients results in less pain relief from knee joint surgery [9].

Over the past two decades, the increasing use of opioids for chronic musculoskeletal pain in the US [10] and Europe [11] has been a serious public health problem. A study using the Medicare Current Beneficiary Survey in the US showed that there was a significant increase in opioid prescribing between 2003 and 2009, resulting in 40% of knee OA patients receiving an opioid in 2009 [12]. However, a more recent study from 2007 to 2014 showed that 15.9% of patients with knee OA were prescribed opioids for their condition and that yearly rates of prescription were fairly stable over this period [13]. Factors related to opioid use have previously been suggested as age, sex, functional limitation, poor self-reported health status, comorbidities, and healthcare environment [12–18]. However, several previous studies examined mixed populations, such as patients with any site of OA or patients with non-cancer pain, and obtained inconsistent results for several factors. In addition, a recent study in the US revealed substantial statewide variation in the prescription of opioids in OA patients [19].

Until now, the prevalence of opioid use as an early treatment option for knee OA has not been much studied. Early opioid use to alleviate pain in knee OA may be related to a long-term use of opioids, because knee OA can cause persistent and chronic pain. Therefore, we conducted a retrospective observational study using Korean national claim data to investigate how many opioid prescriptions were given as early treatment for knee OA and to examine factors related to opioid use for knee OA patients. We hypothesized that knee OA patient’s demographics and comorbidities would be related to early opioid use.

## Methods

### Data source

The Health Insurance Review and Assessment (HIRA) database of Korea, which covers 50 million people, or virtually the whole population of Korea, is a national claim database. The Korean national health insurance system offers universal access for most residents to healthcare through the National Health Insurance (NHI) system and through the Medical Aid program for lower income groups [20]. This database contains individual beneficiary information, as well as healthcare service information, including diagnoses, procedures, prescriptions, and tests. We used the full dataset of HIRA between the years 2011 to 2015.

### Population and study design

#### Identification of knee OA patients

**Fig. 1:**
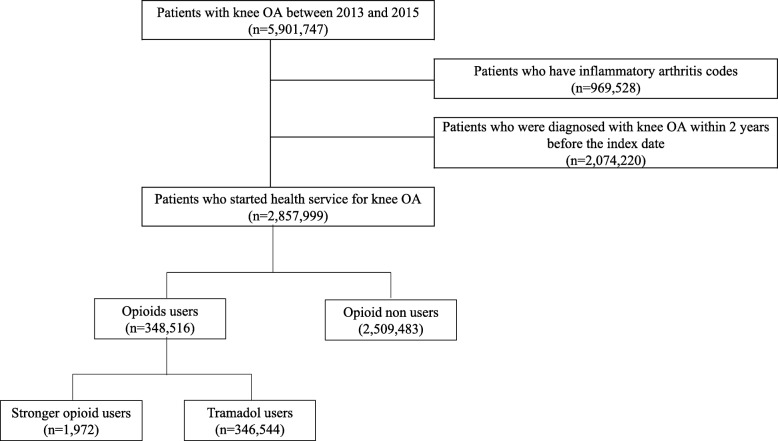
Patient selection flow chart

Knee OA patients between 2013 and 2015 were identified using the operational definition reported in our previous study [21]. Among them, patients with inflammatory arthritis codes such as rheumatoid arthritis, ankylosing spondylitis, and psoriatic arthritis were excluded. We then identified the date of the first claim for knee OA for each patient between 2013 and 2015 and defined it as the index date. We further selected patients without any claim for knee OA for 2 years before the index date (Fig. 1).

#### Definition of opioids and early opioid users

To identify opioids used for early treatment of knee OA, we determined opioid use at the index date and classified patients according to tramadol users and stronger opioid users (hydromorphone, morphine, hydrocodone, oxycodone, pethidine, fentanyl, sufentanil, remifentanil, pentazocine, buprenorphine, butorphanol, nalbuphine, tapentadol, codeine, and dihydrocodeine) (Fig. 1).

#### Factors for the early use of opioids

Demographic characteristics such as age, sex, and type of institution or department related to the opioid prescription were determined at the index date. Medications combined with opioids were also investigated at the index date. Comorbidities were detected based on the Elixhauser comorbidity index [22], which was calculated using ICD 10 codes within 1 year preceding the knee OA index date, because the existing comorbidities could be considered when a physician makes a prescription for opioids in knee OA patients (Additional file 1: Figure S1). In addition, common musculoskeletal diseases related to chronic pain such as low back pain, intervertebral disc disorder, spinal stenosis, and fibromyalgia were also determined based on ICD 10 codes. We performed a sensitivity analysis involving patients without malignancies, because the opioid use to control malignancy pain can influence opioid use for knee OA. In addition, we performed a subgroup analysis of opioid use for 1 year by regular users, defined as patients with medication possession ratios (MPRs) ≥ 75%.

### Statistical analysis

We assessed baseline characteristics of three groups: opioid non-users, tramadol users, or stronger opioid users. Categorical variables are presented as the frequency with percentage, and continuous variables are presented as mean with standard deviation (SD). A multinomial model was constructed to examine factors related to tramadol users or stronger opioid users compared to opioid non-users. All demographic factors and comorbidities that were significant in the crude model, as prespecified in the previous paragraph, were included in the multinomial model. All factors were controlled in the same model. All analyses were performed using SAS 9.2 (SAS Institute, Cary, NC).

### Ethical considerations

This study was determined to be exempt from IRB review by our university hospital IRB (IRB file No. HYUN 2016-06-008-002) because we used the existing, publicly available data, and subject information could not be identified directly through identifiers linked to the subjects.

## Results

### Prevalence of opioid users in knee OA patients and their characteristics

Among all knee OA patients, 348,516 patients (12.2%) were treated with opioids. Most (*n* = 346,544, 12.1%) were tramadol users and only 0.07% (*n* = 1972) were stronger opioid users. Opioids for knee OA patients were prescribed mainly by orthopedic surgeons (70.1%) followed by internists (9.7%), including rheumatologists.

When we compared patient characteristics according to the early use of opioids, opioid users were slightly older (64.2 vs. 64.0 years old, *p* < 0.01) and more likely to be male (44.0% vs. 37.8%, *p* < 0.01) than opioid non-users. The Elixhauser comorbidity index score was higher in opioid users than opioid non-users (4.51 ± 6.36 vs. 4.43 ± 6.23, *p* < 0.01). Differences in comorbidities and concomitant medications between the two groups are presented in Table 1.

**Table 1:** Baseline characteristics of knee OA patients according to the use of opioids as the first analgesic treatment

Variable	Opioid users^§^			Opioid non-users
	Total (*n* = 348,516)	Tramadol (*n* = 346,544)	Stronger opioids (*n* = 1972)	(*n* = 2,509,483)
	*N* (%)	*N* (%)	*N* (%)	*N* (%)
Age, years (mean ± SD)	64.2 ± 9.9	64.1 ± 9.8	68.3 ± 11.0	64.0 ± 9.8
50–59	137,190 (39.4)	136,658 (39.4)	532 (27.0)	997,164 (39.7)
60–69	107,022 (30.7)	106,485 (30.7)	537 (27.2)	781,375 (31.1)
≥ 70	104,304 (29.9)	103,401 (9.8)	903 (45.8)	730,944 (29.1)
Sex (female)	195,021 (56.0)	193,960 (56.0)	1061 (53.8)	1,562,071 (62.2)
Health insurance				
National insurance	323,934 (92.9)	322,308 (93.0)	1626 (82.5)	2,352,280 (93.7)
Medical aid	22,104 (6.3)	21,809 (6.3)	295 (15.0)	136,862 (5.5)
Veterans	2478 (0.7)	2427 (0.7)	51 (2.6)	20,341 (0.8)
Type of institution^†^				
Tertiary hospital	5597 (1.6)	5119 (1.5)	478 (24.2)	33,123 (1.3)
General hospital	29,424 (8.4)	28,395 (8.2)	1029 (52.2)	182,805 (7.3)
Hospital	54,905 (15.8)	54,519 (15.7)	386 (19.6)	336,196 (13.4)
Clinic	253,698 (72.8)	253,624 (73.2)	74 (3.8)	1,684,123 (67.1)
Oriental medical hospital	74 (0.0)	74 (0.0)	–	222,631 (8.9)
Others	4818 (1.4)	4813 (1.4)	5 (0.3)	50,605 (2.0)
Type of department^†^				
Internal medicine	33,778 (9.7)	33,420 (9.6)	358 (18.2)	219,877 (8.8)
Orthopedics	244,305 (70.1)	243,184 (70.2)	1121 (56.8)	1,588,219 (63.3)
Rehabilitation	5480 (1.6)	5424 (1.6)	56 (2.8)	46,811 (1.9)
Oriental medicine	–	–	–	226,250 (9.0)
Others	64,953 (18.6)	64,516 (18.6)	437 (22.2)	428,326 (17.1)
Elixhauser score	4.5 ± 6.4	4.5 ± 6.3	10.3 ± 10.6	4.4 ± 6.2
Comorbidity^††^				
Congestive heart failure	16,502 (4.7)	16,294 (4.7)	208 (10.5)	112,668 (4.5)
Cardiac arrhythmias	12,515 (3.6)	12,364 (3.6)	151 (7.7)	92,539 (3.7)
Peripheral vascular disorders	54,070 (15.5)	53,668 (15.5)	402 (20.4)	356,803 (14.2)
Hypertension, uncomplicated	158,773 (45.6)	157,611 (45.5)	1162 (58.9)	1,125,517 (44.9)
Hypertension, complicated	17,426 (5.0)	17,263 (5.0)	163 (8.3)	123,765 (4.9)
Paralysis	3317 (1.0)	3232 (0.9)	85 (4.3)	22,838 (0.9)
Other neurological disorders	14,069 (4.0)	13,860 (4.0)	209 (10.6)	97,410 (3.9)
Chronic pulmonary disease	104,644 (30.0)	103,822 (30.0)	822 (41.7)	734,749 (29.3)
Diabetes, uncomplicated	67,736 (19.4)	67,064 (19.4)	672 (34.1)	478,114 (19.1)
Diabetes, complicated	37,415 (10.7)	37,046 (10.7)	369 (18.7)	259,686 (10.3)
Hypothyroidism	14,665 (4.2)	14,546 (4.2)	119 (6.0)	120,335 (4.8)
Renal failure	5111 (1.5)	5023 (1.4)	88 (4.5)	36,877 (1.5)
Liver disease	73,216 (21.0)	72,548 (20.9)	668 (33.9)	522,157 (20.8)
Peptic ulcer disease excluding bleeding	80,772 (23.2)	80,162 (23.1)	610 (30.9)	542,506 (21.6)
Metastatic cancer	1614 (0.5)	1386 (0.4)	228 (11.6)	10,203 (0.4)
Solid tumor without metastasis	17,432 (5.0)	17,016 (4.9)	416 (21.1)	136,534 (5.4)
Weight loss	3000 (0.9)	2847 (0.8)	153 (7.8)	19,827 (0.8)
Fluid and electrolyte disorders	13,631 (3.9)	13,341 (3.8)	290 (14.7)	88,210 (3.5)
Deficiency anemia	15,807 (4.5)	15,513 (4.5)	294 (14.9)	115,735 (4.6)
Alcohol abuse	7674 (2.2)	7593 (2.2)	81 (4.1)	48,089 (1.9)
Depression	41,634 (11.9)	41,113 (11.9)	521 (26.4)	277,119 (11.0)
Musculoskeletal disorders				
Low back pain	187,194 (53.7)	185,990 (53.7)	1204 (61.1)	1,257,774 (50.1)
Intervertebral disc disorder	69,342 (19.9)	68,789 (19.9)	553 (28.0)	413,702 (16.5)
Spinal stenosis	74,070 (21.3)	73,357 (21.2)	713 (36.2)	405,241 (16.1)
Fibromyalgia	6576 (1.9)	6532 (1.9)	44 (2.2)	39,799 (1.6)
Medication^†^				
NSAIDs	246,933 (70.9)	245,578 (70.9)	1355 (68.7)	1,492,548 (59.5)
Nonselective	233,275 (66.9)	232,418 (67.1)	857 (43.5)	1,372,520 (54.7)
Cox-2 inhibitor use	14,793 (4.2)	14,175 (4.1)	618 (31.3)	123,006 (4.9)
Acetaminophen	7909 (2.3)	7589 (2.2)	320 (16.2)	143,820 (5.7)
SYSADOA^*^	78,876 (22.6)	78,593 (22.7)	283 (14.4)	554,849 (22.1)
Steroid	15,561 (4.5)	15,443 (4.5)	118 (6.0)	59,245 (2.4)
GI-protective agents^¶^	217,241 (62.3)	215,953 (62.3)	1288 (65.3)	976,102 (38.9)

*OA* osteoarthritis, *NSAIDs* nonsteroidal anti-inflammatory drugs, *SYSADOA* symptomatic slow-acting drugs for OA, *GI* gastrointestinal

^**†**^Type of institution, department, and medication were determined at the index date

^**††**^Comorbidities with more than 3% prevalence

^*^SYSADOA includes diacerein, avocado, glucosamine, sadenin, chondroitin sulfate, and herbal SYSADOA such as Layla, Joins, and Shinbaro

^**¶**^GI protective agents include proton pump inhibitors, H2 blockers, rebamipide, teprenone, and *Artemisia asiatica*

^§^Opioid user includes combination products of acetaminophen

### Factors for early use of opioids in knee OA patients

After classifying opioid users as tramadol or stronger opioid users, we generated a multinomial model that compared the two groups with opioid non-users (Table 2). Several similar factors were associated with early tramadol and stronger opioid use, including male sex (OR 1.28 for tramadol, OR 1.13 for stronger opioids), tertiary hospital (OR 1.04 for tramadol, OR 56.63 for stronger opioids), and depression (OR 1.05 for tramadol, OR 1.46 for stronger opioids), low back pain (OR 1.13 for tramadol, OR 1.30 for stronger opioids), intervertebral disc disorder (OR 1.11 for tramadol, OR 1.40 for stronger opioids), and spinal stenosis (OR 1.27 for tramadol, OR 1.55 for stronger opioids). Patients supported by national insurance (OR 0.94 for tramadol, OR 0.73 for stronger opioids) and rehabilitation visits compared to internal medicine (OR 0.81 for tramadol, OR 0.30 for stronger opioids) were associated with lower opioid use. However, the most recent year of use (2015) was associated with less tramadol use (OR 0.98, 95% CI 0.97–0.99) and more use of stronger opioids (OR 1.40 in 2014, OR 1.14 in 2015). This finding showed the possibility of an increasing tendency to use stronger opioids over tramadol. Patients in their 60s were less likely to receive tramadol than those in their 50s (OR 0.97, 95% CI 0.96–0.98), but those over 70 years old were more likely to take stronger opioids compared to those in their 50s (OR 1.45, 95% CI 1.29–1.63). In terms of comorbidities, myocardial infarction (OR 0.96, 95% CI 0.93–0.99), peptic ulcer disease (PUP) (OR 1.04, 95% CI 1.03–1.05), and fibromyalgia (OR 1.13, 95% CI 1.10–1.16) were the factors associated only with tramadol and not stronger opioids. Diabetes (OR 0.97, 95% CI 0.96–0.98) and malignancy (OR 0.87, 95% CI 0.85–0.88) were associated with less tramadol, but stronger opioids (diabetes [OR 1.21, 95% CI 1.10–1.33] and malignancy [OR 2.91, 95% CI 2.60–3.25]). Dementia (OR 1.24, 95% CI 1.07–1.43) and alcohol abuse (OR 1.39, 95% CI 1.10–1.75) were significantly more associated with stronger opioids. The outcome of the sensitivity analysis in patients without malignancies was similar to that of the main analysis (Additional file 1: Table S2). In the subgroup analysis, we evaluated factors related to regular opioid use MPR of ≥ 75% for knee OA: those in the 60s and 70s were less likely to be regular opioid users than those in the 50s (OR 0.82, 95% CI 0.80–0.83 for the 60s, OR 0.76, 95% CI 0.74–0.77 for the over 70s), and patients with musculoskeletal disease were less likely to use opioids regularly, while patients supported by the national insurance were more likely to use opioids regularly than those receiving medical aid or veterans (OR 1.16, 95% CI 1.13–1.19) (Additional file 1: Table S3).

**Table 2:** Factors related to the use of tramadol or stronger opioids compared with non-use in knee OA patients

Variables	Tramadol (OR, 95% CI)	Stronger opioids (OR, 95% CI)
Year		
2013 (reference)	1	1
2014	1.00 (0.99–1.01)	1.40 (1.26–1.56)
2015	0.98 (0.97–0.99)	1.14 (1.02–1.28)
Age		
50–59 (reference)	1	1
60–69	0.97 (0.96–0.98)	0.99 (0.87–1.12)
≥ 70	1.01 (1.00–1.02)	1.45 (1.29–1.63)
Sex		
Female (reference)	1	1
Male	1.28 (1.27–1.29)	1.13 (1.03–1.24)
Health insurance		
Medical aid/veterans (reference)	1	1
National insurance	0.94 (0.93–0.96)	0.73 (0.64–0.83)
Type of institution		
Hospital/clinic (reference)	1	1
Tertiary hospital	1.04 (1.01–1.07)	59.63 (52.27–68.04)
General hospital	0.97 (0.96–0.99)	21.19 (18.90–23.75)
Others	0.12 (0.12–0.13)	0.06 (0.02–0.13)
Type of department		
Internal medicine (reference)	1	1
Orthopedics	1.00 (0.99–1.01)	0.46 (0.41–0.52)
Rehabilitation	0.81 (0.79–0.84)	0.30 (0.23–0.40)
Others	0.95 (0.94–0.97)	0.87 (0.76–1.01)
Myocardial infarction	0.96 (0.93–0.99)	1.12 (0.84–1.51)
Congestive heart failure	1.01 (1.00–1.03)	1.08 (0.93–1.26)
Peripheral vascular disease	1.04 (1.03–1.05)	1.07 (0.95–1.20)
Dementia	0.98 (0.97–1.00)	1.24 (1.07–1.43)
Chronic pulmonary disease	1.00 (0.99–1.01)	1.16 (1.06–1.28)
Peptic ulcer disease	1.03 (1.02–1.04)	1.09 (0.99–1.20)
Diabetes	0.97 (0.96–0.98)	1.21 (1.10–1.33)
Any malignancy	0.87 (0.85–0.88)	2.91 (2.60–3.25)
Alcohol abuse	1.01 (0.99–1.04)	1.39 (1.10–1.75)
Depression	1.05 (1.04–1.06)	1.46 (1.31–1.62)
Low back pain	1.13 (1.12–1.13)	1.30 (1.18–1.43)
Intervertebral disc disorder	1.11 (1.10–1.12)	1.40 (1.26–1.56)
Spinal stenosis	1.27 (1.26–1.28)	1.55 (1.40–1.72)
Fibromyalgia	1.13 (1.10–1.16)	0.93 (0.69–1.26)

*OA* osteoarthritis, *OR* odds ratio, *CI* confidence interval

## Discussion

In Korea, 12.2% of knee OA patients were treated with opioids as an early treatment option, and tramadol was more commonly used than stronger opioids. Male sex as well as having comorbidities such as depression or musculoskeletal diseases was a patient factor associated with the early use of opioids in knee OA patients. In the most recent year of use (2015), patients were less likely to take tramadol and more likely to take stronger opioids. Compared with age in the 50s, age in the 60s was associated with less tramadol use, while age over 70 years was more associated with stronger opioid use.

The opioid use in Korean patients with knee OA was less than the overall opioid use in other Western countries. The prevalence of opioid use was 40% in the US patients with knee OA [12] and nearly a third of Canadian pre-surgical patients with end-stage knee OA [17]. Opioid use as an early treatment in Korea was similar to that of 12% in Swedish patients with knee or hip OA, but the type of opioids differed from those in Sweden. In Sweden, the most commonly used opioids are stronger, amounting to 17% of OA patients overall, followed by weak opioids, amounting to 9% of OA patients [18]. It is difficult to draw a direct comparison between the results from different countries, because drug utilization may vary depending on the severity of knee OA and the time frame of the investigation. However, it is obvious that the use of stronger opioids was low in Korean patients with knee OA. The low frequency of stronger opioids in Korea may be related to physician reluctance to prescribe stronger opioids for knee OA patients or high accessibility of medical care in Korea. On the other hand, tramadol can be suggested for patients who do not respond to other medications or cannot take other medication, according to a clinical practice guideline for the treatment of knee OA approved by the Korean Knee Society [23]. In addition, formulations of tramadol with acetaminophen and its various generic drugs may have also contributed to the observed high frequency of tramadol. However, this speculation needs further investigation.

Previous studies about the association of age and sex with opioid use show inconsistent results. One study in the US found that older age and female sex were associated with opioid use [15], while another US study found an increased use among younger patients and women [12]. Meanwhile, a Canadian survey in older patients with hip and knee OA observed no association between opioid use and age or sex [16]. These differences are likely attributable to the differential perception of the safety of opioid treatment in different geographic regions. In our study, males were more likely to be treated with opioids, patients in their 60s were less likely to use tramadol, and patients in their 70s were more likely to use stronger opioids compared to patients in their 50s. The frequent use of stronger opioids in elderly males more than 70 years old and the increase in stronger opioid use depending on periods of use are noteworthy. Elderly males may be more likely to experience musculoskeletal diseases or alcohol abuse, and these combined diseases may result in association with stronger opioids. However, careful monitoring should be considered during the opioid use in elderly patients, because of greater exposure to cognitive impairment and fall injuries when they are on opioids. In addition, for men, opioid-related hypogonadism was associated with lower bone mineral density and adverse cardiovascular outcomes [24].

In addition, patients supported by national insurance, compared with medical aid or veterans, were less likely to use opioids. This finding was consistent with previous studies showing that low economic status is associated with opioid use [25, 26]. Treatment at tertiary hospitals was also a factor related to opioid use, possibly implying that patients who visit a tertiary hospital suffer from more intractable stages of knee OA compared to patients who visit hospitals/clinics. The department of rehabilitation was less likely to prescribe tramadol or stronger opioids, and orthopedics was less likely to prescribe stronger opioids. These differences might be explained by patient preference: patients who want to find non-pharmacological treatment options would more likely visit the department of rehabilitation or orthopedics, rather than the department of rheumatology.

The observed increased risk of opioid use for patients with comorbidities of musculoskeletal disease [12], COPD [12], and depression [12, 14, 17, 27] was consistent with previous studies. Alcohol abuse and dementia were also significant factors associated with the use of stronger opioids. It is unclear whether patients with such mental comorbidities have a lower pain threshold and diminished responsiveness to opioids [28, 29] or perhaps lower patient empowerment. However, a previous study showed that mental health conditions, substance dependence and abuse, and preexisting pain disorders increase the risk for prolonged opioid use among opioid-naïve patients [30]. Therefore, physicians need to prescribe opioids carefully in patients with mental comorbidities or preexisting pain disorders, because the risk of long-term use can increase in such patients.

The strength of the present study was that it was a large population-based study using a Korean nationwide database that covers all drugs prescribed to the overall population. The Korean insurance system is a single-payer, universal, and compulsory healthcare system. The reimbursement standard of the national Health Insurance Review and Assessment Service applies to all health providers. This use of comprehensive and longitudinal data avoided selection bias and included all opioid use in knee OA patients. In addition, we selected early treatment for knee OA based on a validated definition. Therefore, this study presented information about early opioid treatment in knee OA patients that has been rarely presented until now.

There were several limitations to this population-based study. First, we did not score the disease stage, pain-intensity, or functional disability due to limitations of the data source. In the opioid users, the prevalence of NSAIDs and SYSADOA prescriptions was higher than in the opioid non-users. Opioid non-users may not use medication for pain because their pain is mild, or they may use oriental medicine or alternative treatments that are not covered by our national insurance. Studies are needed using appropriate data sources to evaluate the use of health functional foods and acupuncture by knee OA patients that do not take pain medication. In addition, we were unable to determine the reason for opioid use. Therefore, we considered several chronic pain-related conditions to evaluate factors related to opioid use. Second, we did not evaluate the safety or outcomes of each opioid, particularly tramadol. Although tramadol is a weak opioid, further study is needed to evaluate long-term safety and outcomes. Third, we could not examine incident knee OA patients, because it was difficult to identify early-stage knee OA patients in the claim database since non-symptomatic early-stage knee OA patients might not need medication. Instead of trying to extract incident patients with knee OA, we restricted our cohort to knee OA patients who had not accessed health services over the 2 previous years to identify patients without prior treatment for knee OA and for whom opioids were an early treatment option. However, some patients with advanced knee OA who had no prior treatment or had received alternative treatments may have been included. Therefore, it is possible that we overestimated the prevalence of the early use of opioids. Fourth, even though we performed a subgroup analysis of patients with regular opioid use, defined by an MPR ≥ 75%, we could not evaluate treatment patterns after the index date, which may vary. Future work on the prescription trajectories of opioids will be needed to determine the actual regular use of opioids. Lastly, factors with odds ratios close to 1 in our study using a large population database should be interpreted carefully.

There is currently a lack of consensus from professional medical organizations around the appropriate use of opioid medications in OA. Our findings about current patterns of opioid use as an early treatment option for knee OA and related factors provide useful information for making appropriate recommendations.

## Conclusion

In Korea, 12.2% of knee OA patients were treated with opioids as an early treatment option, and tramadol was more commonly used than stronger opioids. Male sex as well as having comorbidities such as depression or musculoskeletal diseases was a patient factor associated with the early use of opioids in knee OA patients.

## Supplementary information

**Additional file 1: Figure S1.** Study design. **Table S1.** Factors for early opioid use compared with opioid non-use in knee OA patients, **Table S2.** Factors associated with early opioid use compared with opioid non-use in knee OA patients without malignancies^*,^ **Table S3.** Factors associated with regular opioid use (MPR ≥75%) compared with opioid non-use in knee OA patients^*^.

## Data Availability

The datasets used and/or analyzed during the current study are available from the corresponding author on reasonable request.

## Abbreviations

OA: Osteoarthritis
US: United States
HIRA: Health Insurance Review and Assessment
SD: Standard deviation
