# Supplementary material for: Factors related to the use of opioids as early treatment in patients with knee osteoarthritis
Source: Arthritis Res Ther. 2019 Nov 4;21:222. doi: 10.1186/s13075-019-2004-x (PMC6827242; doi:10.1186/s13075-019-2004-x)
Supplement: Supplementary file 1 — Additional file 1: Figure S1. Study design. Table S1. Factors for early opioid use compared with opioid non-use in knee OA patients, Table S2. Factors associated with early opioid use compared with opioid non-use in knee OA patients without malignancies*, Table S3. Factors associated with regular opioid use (MPR ≥75%) compared with opioid non-use in knee OA patients*. [file 13075_2019_2004_MOESM1_ESM.docx]

Figure S1. Study design

**Index data of initiation treatment for knee OA**

between 2013 and 2015

Comorbidities were detected during 12 months before index date

Baseline demographics, type of institution and type of department were detected at index date

Washout period of 24 months for knee OA claims

Table S1. Factors for early opioid use compared with opioid non-use in knee OA patients^*^

| Variable | Opioid use (OR, 95% CI) |
| --- | --- |
| Year |  |
| 2013 (reference) | 1 |
| 2014 | 1.00 (0.99 - 1.01) |
| 2015 | 0.98 (0.97 - 0.99) |
| Age |  |
| 50-59 (reference) | 1 |
| 60-69 | 0.97 (0.96 - 0.98) |
| ≥70 | 1.02 (1.01 - 1.03) |
| Sex |  |
| Female (reference) | 1 |
| Male | 1.28 (1.27 - 1.29) |
| Health insurance |  |
| Medical aid/Veterans (reference) | 1 |
| National insurance | 0.94 (0.93 - 0.95) |
| Type of institution |  |
| Clinic (reference) | 1 |
| Tertiary hospital | 1.15 (1.11 - 1.18) |
| General hospital | 1.02 (1.00 - 1.03) |
| Hospital | 1.07 (1.06 - 1.08) |
| Others | 0.13 (0.12 - 0.13) |
| Type of department |  |
| Internal medicine (reference) | 1 |
| Orthopedics | 0.99 (0.97 - 1.00) |
| Rehabilitation | 0.80 (0.77 - 0.82) |
| Others | 0.95 (0.93 - 0.96) |
| Myocardial infarction | 0.96 (0.93 - 0.99) |
| Congestive heart failure | 1.02 (1.00 - 1.03) |
| Peripheral vascular disease | 1.04 (1.03 - 1.05) |
| Dementia | 0.98 (0.97 - 1.00) |
| Chronic pulmonary disease | 1.00 (0.99 - 1.01) |
| Peptic ulcer disease | 1.03 (1.02 - 1.04) |
| Diabetes | 0.97 (0.97 - 0.98) |
| Any malignancy | 0.88 (0.87 - 0.90) |
| Alcohol abuse | 1.01 (0.99 - 1.04) |
| Depression | 1.05 (1.04 - 1.06) |
| Low back pain | 1.13 (1.12 - 1.13) |
| Intervertebral disc disorder | 1.11 (1.10 - 1.12) |
| Spinal stenosis | 1.27 (1.26 - 1.28) |
| Fibromyalgia | 1.13 (1.10 - 1.16) |

OA: osteoarthritis, OR: odds ratio, CI: confidence interval

^*^Multivariate logistic regression analysis

Table S2. Factors associated with early opioid use compared with opioid non-use in knee OA patients without malignancies^*^

| Variable | Opioid use (OR, 95% CI) |
| --- | --- |
| Year |  |
| 2013 (reference) | 1 |
| 2014 | 1.00 (0.99 - 1.01) |
| 2015 | 0.98 (0.97 - 0.99) |
| Age |  |
| 50-59 (reference) | 1 |
| 60-69 | 0.97 (0.96 - 0.98) |
| ≥70 | 1.02 (1.01 - 1.02) |
| Sex |  |
| Female (reference) | 1 |
| Male | 1.28 (1.27 - 1.29) |
| Health insurance |  |
| Medical aid/Veterans (reference) | 1 |
| National insurance | 0.93 (0.92 - 0.95) |
| Type of institution |  |
| Clinic (reference) | 1 |
| Tertiary hospital | 1.10 (1.07 - 1.14) |
| General hospital | 1.01 (1.00 - 1.02) |
| Hospital | 1.07 (1.06 - 1.08) |
| Others | 0.13 (0.12 - 0.13) |
| Type of department |  |
| Internal medicine (reference) | 1 |
| Orthopedics | 1.01 (0.99 - 1.02) |
| Rehabilitation | 0.82 (0.80 - 0.85) |
| Others | 0.96 (0.95 - 0.98) |
| Myocardial infarction | 0.95 (0.92 - 0.98) |
| Congestive heart failure | 1.02 (1.00 - 1.03) |
| Peripheral vascular disease | 1.04 (1.03 - 1.05) |
| Dementia | 0.99 (0.97 - 1.00) |
| Chronic pulmonary disease | 1.00 (0.99 - 1.01) |
| Peptic ulcer disease | 1.03 (1.02 - 1.04) |
| Diabetes | 0.98 (0.97 - 0.98) |
| Alcohol abuse | 1.01 (0.99 - 1.04) |
| Depression | 1.05 (1.04 - 1.06) |
| Low back pain | 1.12 (1.12 - 1.13) |
| Intervertebral disc disorder | 1.11 (1.10 - 1.13) |
| Spinal stenosis | 1.27 (1.26 - 1.29) |
| Fibromyalgia | 1.14 (1.11 - 1.17) |

OA: osteoarthritis, OR: odds ratio, CI: confidence interval

^*^Multivariate logistic regression analysis

Table S3. Factors associated with regular opioid use (MPR ≥75%) compared with opioid non-use in knee OA patients^*^

| Variable | Opioid use (OR, 95% CI) |
| --- | --- |
| Year |  |
| 2013 (reference) | 1 |
| 2014 | 1.00 (0.99 - 1.02) |
| 2015 | 1.00 (0.99 – 1.02) |
| Age |  |
| 50-59 (reference) | 1 |
| 60-69 | 0.82 (0.80 - 0.83) |
| ≥70 | 0.76 (0.74 – 0.77) |
| Sex |  |
| Female (reference) | 1 |
| Male | 1.07 (1.05 - 1.09) |
| Health insurance |  |
| Medical aid/Veterans (reference) | 1 |
| National insurance | 1.16 (1.13 – 1.19) |
| Type of institution |  |
| Clinic (reference) | 1 |
| Tertiary hospital | 4.71 (4.34 – 5.11) |
| General hospital | 2.81 (2.72 – 2.89) |
| Hospital | 1.89 (1.85 - 1.93) |
| Others | 1.39 (1.31 – 1.48) |
| Type of department |  |
| Internal medicine (reference) | 1 |
| Orthopedics | 0.84 (0.82 – 0.86) |
| Rehabilitation | 0.82 (0.77 - 0.88) |
| Others | 0.85 (0.82 - 0.87) |
| Myocardial infarction | 0.97 (0.90 – 1.04) |
| Congestive heart failure | 1.03 (1.00 - 1.07) |
| Peripheral vascular disease | 0.93 (0.92 – 0.95) |
| Dementia | 1.26 (1.21 - 1.31) |
| Chronic pulmonary disease | 0.95 (0.94 – 0.97) |
| Peptic ulcer disease | 0.97 (0.96 – 0.99) |
| Diabetes | 1.01 (0.99 – 1.02) |
| Any malignancy | 1.11 (1.07 – 1.15) |
| Alcohol abuse | 0.96 (0.91 - 1.01) |
| Depression | 1.00 (0.98 - 1.03) |
| Low back pain | 0.88 (0.86 – 0.89) |
| Intervertebral disc disorder | 0.94 (0.92 – 0.96) |
| Spinal stenosis | 0.92 (0.90 – 0.94) |
| Fibromyalgia | 0.92 (0.87 – 0.97) |

MPR: medication possession ratios, OA: osteoarthritis, OR: odds ratio, CI: confidence interval

^*^ Multivariate logistic regression analysis was performed.
